# Dengue and chikungunya among outpatients with acute undifferentiated fever in Kinshasa, Democratic Republic of Congo: A cross-sectional study

**DOI:** 10.1371/journal.pntd.0007047

**Published:** 2019-09-05

**Authors:** Sam Proesmans, Freddy Katshongo, John Milambu, Blaise Fungula, Hypolite Muhindo Mavoko, Steve Ahuka-Mundeke, Raquel Inocêncio da Luz, Marjan Van Esbroeck, Kevin K. Ariën, Lieselotte Cnops, Birgit De Smet, Pascal Lutumba, Jean-Pierre Van geertruyden, Veerle Vanlerberghe

**Affiliations:** 1 University of Antwerp, Antwerp, Belgium; 2 Institut Supérieur des Techniques Médicales, Kinshasa, Democratic Republic of Congo; 3 Centre Hospitalier Lisungi, Kinshasa, Democratic Republic of Congo; 4 Université de Kinshasa, Kinshasa, Democratic Republic of Congo; 5 Institut National de Reserche Biomédicale, Kinshasa, Democratic Republic of Congo; 6 Institute of Tropical Medicine, Antwerp, Belgium; Mahidol Univ, Fac Trop Med, THAILAND

## Abstract

**Background:**

Pathogens causing acute fever, with the exception of malaria, remain largely unidentified in sub-Saharan Africa, given the local unavailability of diagnostic tests and the broad differential diagnosis.

**Methodology:**

We conducted a cross-sectional study including outpatient acute undifferentiated fever in both children and adults, between November 2015 and June 2016 in Kinshasa, Democratic Republic of Congo. Serological and molecular diagnostic tests for selected arboviral infections were performed on blood, including PCR, NS1-RDT, ELISA and IFA for acute, and ELISA and IFA for past infections.

**Results:**

Investigation among 342 patients, aged 2 to 68 years (mean age of 21 years), with acute undifferentiated fever (having no clear focus of infection) revealed 19 (8.1%) acute dengue–caused by DENV-1 and/or DENV-2 –and 2 (0.9%) acute chikungunya infections. Furthermore, 30.2% and 26.4% of participants had been infected in the past with dengue and chikungunya, respectively. We found no evidence of acute Zika nor yellow fever virus infections. 45.3% of patients tested positive on malaria Rapid Diagnostic Test, 87.7% received antimalarial treatment and 64.3% received antibacterial treatment.

**Discussion:**

Chikungunya outbreaks have been reported in the study area in the past, so the high seroprevalence is not surprising. However, scarce evidence exists on dengue transmission in Kinshasa and based on our data, circulation is more important than previously reported. Furthermore, our study shows that the prescription of antibiotics, both antibacterial and antimalarial drugs, is rampant. Studies like this one, elucidating the causes of acute fever, may lead to a more considerate and rigorous use of antibiotics. This will not only stem the ever-increasing problem of antimicrobial resistance, but will–ultimately and hopefully–improve the clinical care of outpatients in low-resource settings.

**Trial registration:**

ClinicalTrials.gov NCT02656862.

## Author summary

Malaria remains one of the most important causes of fever in sub-Saharan Africa. However, its share is declining, since the diagnosis and treatment of malaria have improved significantly over the years. Hence leading to an increase in the number of patients presenting with non-malarial fever. Often, obvious clinical signs and symptoms like cough or diarrhea are absent, probing the question: “What causes the fever?” Previous studies have shown that the burden of arboviral infections–like dengue and chikungunya–in sub-Saharan Africa is underestimated, which is why we screened for four common arboviral infections in patients presenting with ‘undifferentiated fever’ at an outpatient clinic in suburban Kinshasa, Democratic Republic of Congo. Among the patients tested, we found that one in ten presented with an acute arboviral infection and that almost one in three patients had been infected in the past. These findings suggest that clinicians should think about arboviral infections more often, thereby refraining from the prescription of antibiotics, a practice increasingly problematic given the global rise of antimicrobial resistance.

## Introduction

Acute fever is one of the main reasons for healthcare seeking worldwide. In tropical settings, and especially sub-Saharan Africa, malaria is the first cause to be ruled out, which is done increasingly so following the World Health Organization’s *testing before treating* policy of 2010 –through microscopic blood slide examination or a rapid diagnostic test (RDT) [1]. Following the introduction of this policy, together with the roll-out of the highly efficacious artemisinin-combination therapy as first-line malaria treatment and efficacious vector control, the overall malaria burden declined over the last decade [1]. Accordingly, clinicians face a relatively higher number of malaria-negative patients for whom they do not have a clear diagnosis [2–4]. In sub-Saharan Africa, where healthcare settings are often resource-limited, healthcare providers face the daunting challenge pinpointing the causing agent of acute fever in an adequate and timely fashion, with little to no diagnostic means other than a malaria-RDT. They mostly rely on history taking and physical examination to determine the focus and cause of infection, of which acute respiratory infection (ARI), gastroenteritis (GE) and urinary tract infection (UTI) are the three most prevalent syndromes reported [4]. However, for some patients presenting with acute fever no focus of infection can be found, thus labeling them as ‘undifferentiated’–knowing that their differential diagnosis is broad, ranging from viral, bacterial, parasitic to fungal infections. Some studies have found that this ‘undifferentiated’ group among fever patients represents 20 to 40% of the grand total [4]. Although viral illnesses are often suspected, both prescription and over-the-counter usage of antimicrobials is rampant in this group globally and their licentious usage in low-resource settings fuels the global burden of antimicrobial resistance [5,6]. More insight in the exact causes of this group of ‘undifferentiated fevers’ may help curb the usage of antimicrobials and improve the clinical care of patients in low-resource settings more broadly [7]. Still, evidence on the causes of ‘undifferentiated acute fever syndromes’ is scarce and is coming almost entirely from inpatient settings. Indeed, to our knowledge only one study in sub-Saharan Africa, namely in Sierra Leone in 2012–2013, looked in a prospective way at the etiologies of acute fever–using RDTs–in patients with self-reported or clinically confirmed fever with a maximum duration of 7 days, finding 5% acute dengue virus (DENV) infection and 39% acute chikungunya virus (CHIKV) or other alphavirus infection [8]. Nonetheless, limited outbreaks and sporadic clinical cases of DENV have been reported over the last 50 years in 22 African countries [9]. Seroprevalence studies have demonstrated DENV IgG-antibodies, indicating past-infection, in 12.5% of study participants in Cameroon, 36% in Burkina Faso and 45% in Nigeria [9], although in other areas seropositivity remained zero [10]. In Tanzania past infection rates are higher, reaching 50.6% in health-facility based studies and 11% in community-based studies [11]. Despite the presence of all four DENV serotypes, severe disease epidemics are rarely reported in Africa [12]. The DENV burden in Africa is, based on modeling, estimated at 16 million symptomatic clinical infections or 16% of the global total [13]. In East-Africa, CHIKV outbreaks and circulation are described, such as in Kenya with a past-infection rate of 67% [14] and the reports of epidemics in 2004 in Kenya [15], in 2013 in Tanzania [16] and in 2018 in Mozambique [17].

These viral vector-borne diseases are also circulating in the Central African region. This is illustrated by CHIKV outbreaks in Kinshasa in both 2000 [18] and 2012 [19] and Brazzaville in 2011 [20], and the 2013 DENV [21] and 2016 yellow fever virus (YFV) outbreak in Angola [22], along with the first Zika virus (ZIKV) case reported in Angola in 2017 [23]. These outbreaks are only possible because the *Aedes* mosquito, the vector of the aforementioned arboviruses, thrives in this region. Furthermore, although not conclusive for vector competence and local transmission capability, alphaviruses (chikungunya) and flaviviruses (species not specified) were demonstrated by RT-PCR in *Aedes* mosquitoes in Kinshasa in 2014 [24].

In the Democratic Republic of Congo (DRC) the circulating pathogens causing uncomplicated acute undifferentiated fever, are unknown [25]. However, outside the above documented epidemics, CHIKV and DENV probably circulate continuously. Of travelers returning from Africa (2007–2012) and attending the outpatient clinic of the Institute of Tropical Medicine in Antwerp, Belgium, 22% of those diagnosed with a CHIKV infection came from DRC [26] and up to that point there was an increasing number of confirmed DENV infections in travelers coming from a large set of African countries, including DRC [27]. In Eastern DRC, a few DENV cases have been found during an outbreak of West-Nile fever in 1998 [28] and between 2003 and 2012 when testing samples negative for YFV [29].

In this study in DRC, we aim to quantify the importance of four major arboviruses as a cause of acute undifferentiated fever. Furthermore, we aim to describe the case presentation and the presence of arbovirus/malaria co-infections, since *Plasmodium falciparum* is still responsible for an estimated 25 million cases nationwide–with 97% of the country being a ‘high transmission’ region, ranking DRC among the top 3 countries in sub-Saharan Africa with the highest malaria burden [30].

## Methods

### Ethics statement

This study was approved by the ethical review boards of the School of Public Health of the University of Kinshasa (DRC), the Institute of Tropical Medicine of Antwerp (Belgium) and the University Hospital of Antwerp (Belgium). The study was registered in a public repository (https://www.clinicaltrials.gov/ct2/show/NCT02656862). Written informed consent was obtained from every adult or–in case of minors–from their caretaker. This study was conducted in full compliance with the principles of the latest amended *Declaration of Helsinki* and of the *International Conference Harmonization* (ICH) guidelines, plus adhering to local laws and regulations.

### Setting

The study took place in Lisungi health center in Pumbu, an area of about 14,000 inhabitants, belonging to the peri-urban health district Mont Ngafula 1, at the southern side of Kinshasa. The climate is tropical with a rainy season between October and May, and a dry season from June to September. The Lisungi health center is the only public health facility in the area, with a medical staff of 40 persons averaging 250 patient encounters per week–which are provided for a small out-of-pocket contribution, as is commonplace throughout DRC. It treats mainly outpatients, but several inpatient beds are available for short time follow-up of more complicated cases. Over the years, around 70% of patients mention fever as the reason for medical care seeking, of whom half tested positive for malaria on RDT (personal communication with Dr. Blaise Fungula). There are no means for other microbiological testing. The Lisungi health center has recently performed a *Good Clinical Laboratory Practice* compliant trial and has been involved in other febrile illness investigations, specifically on malaria [31].

### Study design and participants

The study was designed as a cross-sectional study with prospective patient inclusion. As the proportion of pathogens can change over time, especially for epidemic-prone diseases, we included patients proportionally from November 2015 to June 2016. Only patients of at least 2 years old, presenting at the outpatient department with a history of acute fever (*i*.*e*. ≥ 2 days and ≤ 7 days) or having an axillary temperature of ≥ 37.5°C, were eligible. Patients with any history of an acute injury, trauma or poisoning, suspicion of meningitis/encephalitis, recent hospitalization or women who gave birth in the preceding two weeks, were excluded. Reported recent intake of antimicrobials was not an exclusion criterion, but was recorded accordingly. There were two categories of patients included: the ‘undifferentiated fevers’, with as case definition history of acute fever and without any clear clinical focus of infection and the ‘differentiated fevers’, with a history of fever and with acute respiratory infection (ARI), gastroenteritis (GE) or urinary tract infection (UTI) categorized on clinical grounds. Of the first group, a maximum of 6 patients per day (3 children and 3 adults), and of the second group a maximum of 2 patients per day (1 child and 1 adult) were to be included. The latter category was included given the non-specificity of the signs and symptoms of a possible arboviral infection and in order to estimate the burden of co-infection–which was also the reason not to exclude the confirmed malaria cases based on laboratory analysis. Our main interest was the distribution of viral pathogens among the undifferentiated fevers. To be able to detect the presence of a disease whose prevalence is 5% with a precision of 2.5% at a confidence level of 95%, 290 patients needed to be included. Increased with 10% for incomplete data or loss of biological samples, we came to a minimal sample size of 320.

### Data collection

#### Case report form

For every participant, a pre-tested case report form (CRF) was filled out, based on a standardized clinical history and physical examination by one of the four physicians/clinical officers on the ground, following standard clinical practice. Prior to the start of study, the clinicians received a training on the inclusion algorithm and CRF. Patient management was done according to local guidelines. For every patient the following information was recorded: demographic data (age, sex, residence), past medical history (yellow fever vaccination, intake of antimicrobials), signs and symptoms, duration of fever, medical examination, diagnosis and treatment given.

#### Laboratory analyses

In the on-site laboratory of the Lisungi health center, capillary blood was taken for a microscopic leukocyte count and a Giemsa-stained thin smear. Hematocrit and hemoglobin were determined using a portable spectrophotometer (Hemocontrol, EKF Diagnostics, Barleben, Germany). Malaria and dengue were diagnosed by the HRP2/pLDH RDT SD Bioline Malaria Ag Pf/Pan 05FK60 and RDT SD Bioline Dengue Duo 11FK46, respectively. The latter is an immunochromatographic one-step assay designed to detect both DENV NS1 antigen (present in first week of infection) and IgM/IgG antibodies (detectable from the fifth day of fever onwards) to DENV. HIV was not routinely tested for, given the overall low prevalence in the study area and the unavailability of HIV antiretroviral treatment according to national ethics guidelines in this setting.

All undifferentiated fevers, together with about half of both the malaria positives and the clinically apparent differentiated fevers–identified through random selection, were tested for arbovirus exposure. For this purpose serum and plasma were stored in liquid nitrogen (-196°C) on-site and transported afterwards to the Institut National de Recherche Biomédicale (INRB) in Kinshasa. After six and nine months, the samples were airlifted to the Institute of Tropical Medicine in Antwerp, Belgium, for additional arbovirus specific tests. Molecular tests were done for DENV, CHIKV and YFV on all available samples. For ZIKV, as there was no evidence of its circulation in the area, testing was done on 50 randomly selected undifferentiated fevers and 30 clinically apparent ‘differentiated fevers’ (through the random numbers tool in Microsoft Excel). The real-time reverse transcriptase polymerase-chain reaction (RT-PCR) for DENV, CHIKV, YFV and ZIKV were done with in house PCR protocols currently in use in the reference laboratory of the Institute of Tropical Medicine Antwerp. The IgM and IgG antibodies for DENV and CHIKV were detected using enzyme-linked immunosorbent assays (ELISA: Dengue Virus IgM Capture DxSelectTM and Dengue Virus IgG DxSelectTM from Focus Diagnostics, Cypress, CA, USA and CHIKV ELISA IgM and CHIKV ELISA IgG test from Euroimmun, Lübeck, Germany). An optical density ratio (O.D.) on ELISA IgG or ELISA IgM >1 was considered as a positive ELISA IgG or IgM result, respectively (as indicated in the kit-instructions). An indirect immunofluorescence antibody assay (IFA: anti-CHIKV IFA from Euroimmun, Lübeck, Germany) for both CHIKV IgM and IgG, considered to be more sensitive and specific based on the literature [32–34] and our own experience in the clinical laboratory of the Institute of Tropical Medicine Antwerp, was done on the ELISA positive samples and on a random selection of 20 negative samples. In case of a non-congruent result between ELISA and IFA, a malaria PCR was done to evaluate if cross-reaction between malaria and CHIKV could explain this finding [35]. A selection of IgG DENV ELISA positive samples (all samples with O.D. <1.9 on ELISA IgG DENV and 6 randomly selected samples of the ones with O.D. >1.9) were tested with DENV and YFV plaque reduction neutralization tests (PRNT) to differentiate between past exposure to DENV or YFV (the PRNT does not discriminate natural infection from vaccination). For the relevant references of the PCR-tests performed, see S1 Text.

#### Case definitions

Acute DENV infection was defined as a positive DENV-PCR and/or a positive DENV-IgM-ELISA and/or a positive NS1.

Past DENV infection was defined as a positive DENV-IgG-ELISA in the absence of DENV-NS1, RNA and IgM antibodies.

Acute CHIKV infection was defined as a positive CHIKV-PCR and/or positive CHIKV-IgM by IFA.

Past CHIKV infection was defined as a positive CHIKV-IgG by IFA in the absence of CHIKV RNA and IgM antibodies.

Acute YFV infection was defined as a positive YFV-RT-PCR.

Acute ZIKV infection was defined as a positive ZIKV-RT-PCR.

#### Statistical analysis

Data were entered into an Excel database (Microsoft Corp, Va., USA). Statistical analyses were performed with SPSS Statistics version 23 (IBM, NY, USA). The Pearson Chi-square test was used to determine the association between categorical variables. An alpha level of 0.05 was used for all tests for statistical significance.

## Results

Over the period November 2015 to June 2016, 342 patients were included from whom clinical data and on the spot malaria-RDT results were recorded. Clinical diagnoses were as follows: 70.2% undifferentiated fever, 17.0% ARI, 6.1% GE and 6.7% UTI–all three further labeled as ‘differentiated fevers’. The study population (Table 1) consisted of 183 (53.5%) female participants and 180 (53.1%) were under the age of 18. Only 10 (2.9%) reported having a chronic disease such as diabetes or sickle cell anemia and 50 (14.6%) patients were in need of hospitalization. The reported YFV vaccination rate was as low as 1%. The vast majority (77.8%) of patients presenting in the four first days after the onset of fever, came from the commune Mont Ngafula, and a minority from neighbouring Selembao and Ngaliema–acute and past arbovirus infections were detected in patients from these three communes (Table 2). Before going to the health center, 15.5% of the participants already self-medicated with one or more tablets of an anti-malarial drug. At presentation, malaria RDT for *Plasmodium falciparum* was positive in 155 (45.3%) of the total number of participants (47.9% and 39.2% in undifferentiated and differentiated fever groups, respectively, p = 0.139). However, 300 (87.7%) received an antimalarial treatment, which was significantly more in the undifferentiated (92.1%) than the differentiated fever group (77.5%) (p<0.001). Antibacterial drugs were frequently prescribed: 64.3% of participants received at least one antibacterial drug, significantly more in the differentiated (72.5%) than the undifferentiated group (60.8%) (p = 0.039) and 11.7% received even more than one antibacterial drug (29.4% in the differentiated and 9.6% in the undifferentiated fever group, p<0.001).

**Table 1:** Baseline characteristics of acute undifferentiated fever patients and their arbovirus exposure at Lisungi health center, DRCongo, 11/2015-06/2016 (n = 342).

Characteristics	All participants N = 342 (%)	Recent DENV infection (n = 19) N (%)	Recent CHIKV infection (n = 02) N (%)	Past DENV infection (n = 71) N (%)	Past CHIKV infection (n = 62) N (%)
Age (years) (n = 339):					
- < 5	56 (16.5)	3 (15.8)	0	7 (9.9)	2 (3.3)
- 5–17	124 (36.6)	6 (31.6)	0	10 (14.1)	16 (26.7)
- 18–44	115 (33.9)	8 (42.1)	2 (100)	35 (49.3)	27 (45.0)
- ≥45	44 (13.0)	2 (10.5)	0	19 (26.8)	15 (24.2)
Gender, Female	183 (53.5)	11 (57.9)	2 (100)	36 (50.7)	31 (50.0)
Consulting in June (dry season)	52 (15.2)	10 (52.6)	0 (0)	10 (14.1)	12 (19.4)
Residence:					
- Mont-Ngafula	250 (73.1)	16 (84.2)	2 (100)	49 (69.0)	50 (80.6)
- Ngaliema	32 (9.4)	2(10.5)	0	5 (7.0)	6 (9.7)
- Selembao	56 (16.4)	1 (5.3)	0	17 (23.9)	6 (9.7)
- Other	4 (1.2)	0	0	0	0
Recent travel outside residence commune	10 (2.9)	1 (5.3)	0 (0)	4 (5.6)	1 (1.6)
Reported yellow fever vaccination	4 (1.2)	0 (0)	0 (0)	1 (1.4)	0 (0)

**Table 2:** Clinical characteristics of acute undifferentiated fever patients stratified by DENV and CHIKV infection at Lisungi health center, DRCongo, 11/2015-06/2016.

Characteristics	All participants (n = 342)	Acute DENV cases (n = 19)	Acute CHIKV cases (n = 2)
	N (%)	N (%)	N (%)
**Clinical presentation**			
Presence of chronic disease	10 (2.9)	0 (0)	0
Rash	26 (7.6)	1 (5.3)	0
Muscular pain	127 (37.1)	3 (15.8)	1 (50)
Painful or inflamed joints	168 (49.1)	6 (31.6)	2 (100)
Headache	234 (68.4)	12 (63.2)	1 (50)
Tiredness	172 (50.3)	11 (57.9)	0
Respiratory symptoms (upper and lower tract)	132 (38.6)	5 (26.3)	0
Bleeding	1 (0.3)	0 (0)	0
Nausea	76 (22.2)	3 (15.8)	1 (50)
Gastro-enteral symptoms (Vomiting, diarrhea, jaundice)	81 (23.7)	0 (0)	0
Abdominal pain	66 (19.3)	2 (10.5)	0
Lymphadenopathy	94 (27.5)	1 (5.3)	1 (50)
Hepatomegaly and/or splenomegaly and/or abnormal abdominal palpation	115 (33.6)	1 (5.3)	2 (100)
**Laboratory results**			
Positive malaria rapid diagnostic test	155 (45.3)	6 (31.6)	0
Hematocrit (Median)	40.0	40.5	39
White blood cells count (Median)	4200	4250	3850
White blood cell formula (Median)			
- neutrophils	66	67	66
- lymphocytes	33	31	33.5
- monocytes	0	0	0.5
- eosinophils	0	0.5	0
- basophils	0	0	0
**Diagnosis and treatment**			
Preliminar diagnosis at start of consultation:			
- undifferentiated fever	240 (70.2)	13 (68.4)	1 (50)
- gastroenteritis (GE)	21 (6.1)	0 (0)	0
- urinary tract infection (UTI)	23 (6.7)	1 (5.3)	1 (50)
- respiratory infection (ARI)	58 (17.0)	5 (26.3)	0
Final diagnosis (>1% of grand total):			
- amygdalitis	11 (3.2)	1 (5.3)	1 (50)
- undifferentiated fever	154 (45.0)	12 (63.2)	0
- malaria	155 (45.3)	6 (31.6)	0
Requiring hospitalization	50 (14.6)	0 (0)	0
Received of at least one antibacterial drug	220 (64.3)	11 (57.9)	2 (100)
Received more than one antibacterial drug	40 (11.7)	2 (10.5)	1 (50)
Received antimalarial treatment	300 (87.7)	16 (84.2)	1 (50)

**Fig 1:**
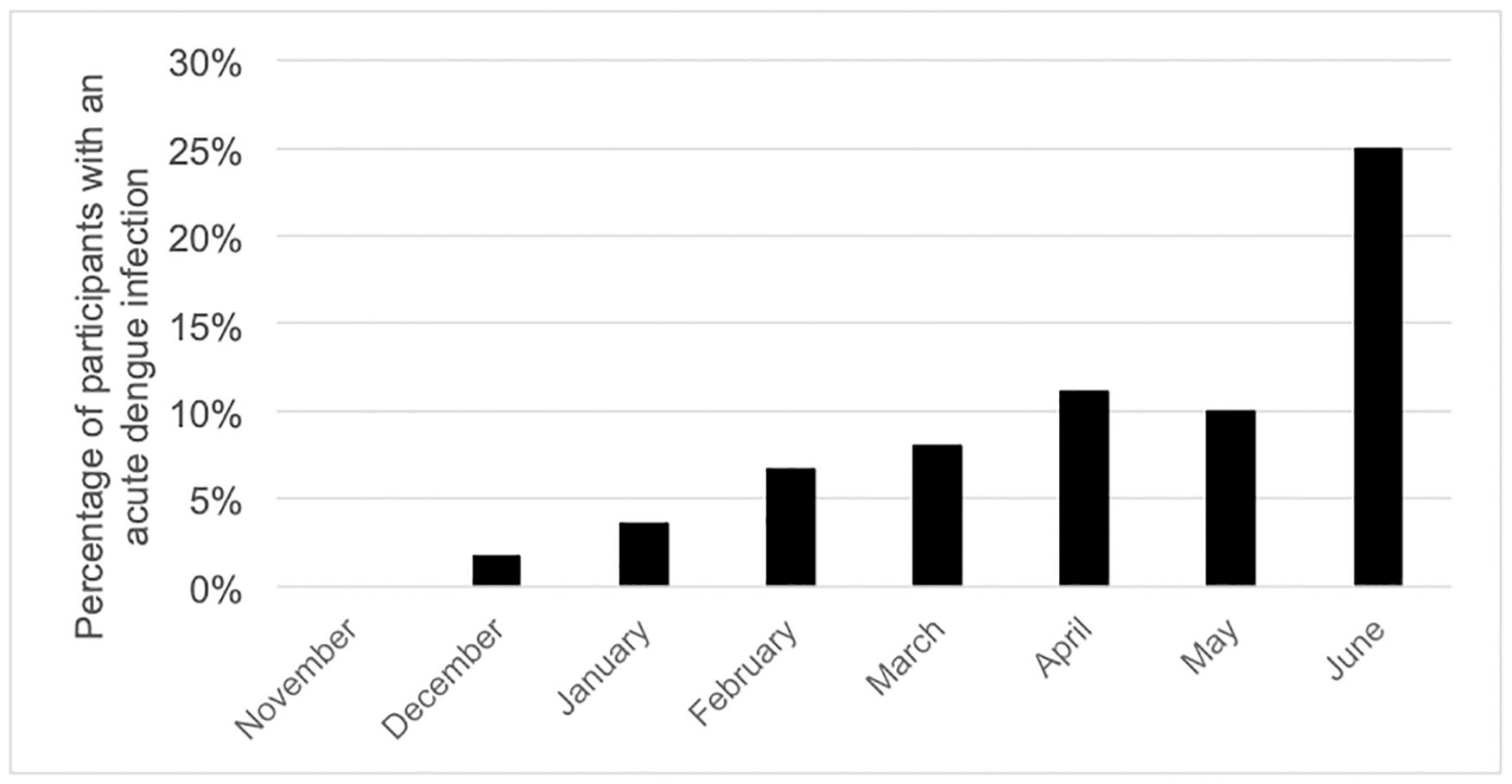
Percentage of participants having an acute dengue infection, Lisungi health center, DRCongo, 2015–2016.

In the 235 participants further tested for arboviruses, 19 (8.1%) fulfilled the criteria of an acute DENV infection, of which 14 were confirmed by RT-PCR. Both serotypes DENV1 and DENV2 were detected (Table 3). Five participants were presumptively infected with DENV, based on the presence of IgM antibodies alone. All NS1 positive patients were RT-PCR positive. In contrast, only two acute CHIKV infections were suspected based on the presence of IgM antibodies. The majority of CHIKV IgM ELISA positive samples was not confirmed by IFA. On these non-congruent samples, PCR to detect *Plasmodium* was performed and revealed an actual malaria infection in 18 of the 22 CHIKV IgM ELISA positive /IgM IFA negative. There was a temporal heterogeneity in the appearance of DENV infections (Fig 1). In June, a dry season month which had less than 25mm of rain (www.infoclimat.fr), there was an increased risk of 6.13 (adjusted OR 95% CI 2.24–17.81) of presenting with acute DENV in comparison to the rainy season (S1 Table). Of the acute DENV and CHIKV cases, 31.6% and 0% had a positive malaria RDT, respectively. When focusing on the malaria negative cases, we observed that 8.7% (13/149) tested positive for acute DENV, 1.3% (2/149) for acute CHIKV, none for acute ZIKV, nor for YFV.

**Table 3:** Proportion of positive results diagnostic laboratory tests for arbovirus infections at Lisungi health center, DRCongo, 11/2015-06/2016 (n = 235, unless otherwise stated).

Virus	RDT (+)	PCR (+)	ELISA(+)	IFA (+)	PRNT(+)
**DENV**	NS1: 4.4%	DEN1: 5.1%	IgM: 3.8%	n.a.	Weak IgG ELISA: 10.0%
	IgM: 1.5%	DEN2: 0.9%	IgG: 34.0%		(n = 19*)
	IgG: 2.5%				
	*(n = 204)*				Strong IgG ELISA.: 66.7%
					(n = 6*)
**CHIKV**	n.a.	0%	IgM: 10.2%	IgM: 0.9%	n.a.
		*(n = 226)*	IgG: 30.6%	(n = 50**)	
				IgG: 27.2%	
				(n = 96**)	
**ZIKV**	n.a.	0%	n.a.	n.a.	n.a.
		*(n = 77)*			
**YFV**	n.a.	0%	n.a.	n.a.	22.2%
		*(n = 227)*			*(n = 9)*

*19 samples with O.D. <1.9 on ELISA IgG DEN; 6 samples randomly selected of the ones with O.D. > 1.9

**samples having positive/grey zone results on Chikungunya ELISA test

RDT: rapid diagnostic test, PCR: polymerase chain reaction, ELISA: enzyme linked immunosorbent assay, IFA: immunofluorescence assay, PRNT: plaque reduction neutralization test, NS1: nonstructural protein 1, IgM: immunoglobulin M, IgG: Immunoglobulin G, n.a.: non applicable

With regard to the clinical presentation of both DENV and CHIKV infections, we found no specific signs or symptoms to be statistically significantly–let alone clinically relevant–associated with acute DENV or CHIKV versus the other febrile patients (S2 Table). None of the acute DENV or CHIKV cases were clinically severe enough to require hospitalization and there was no apparent leucopenia or hemoconcentration (as often seen in severe DENV cases).

**Fig 2:**
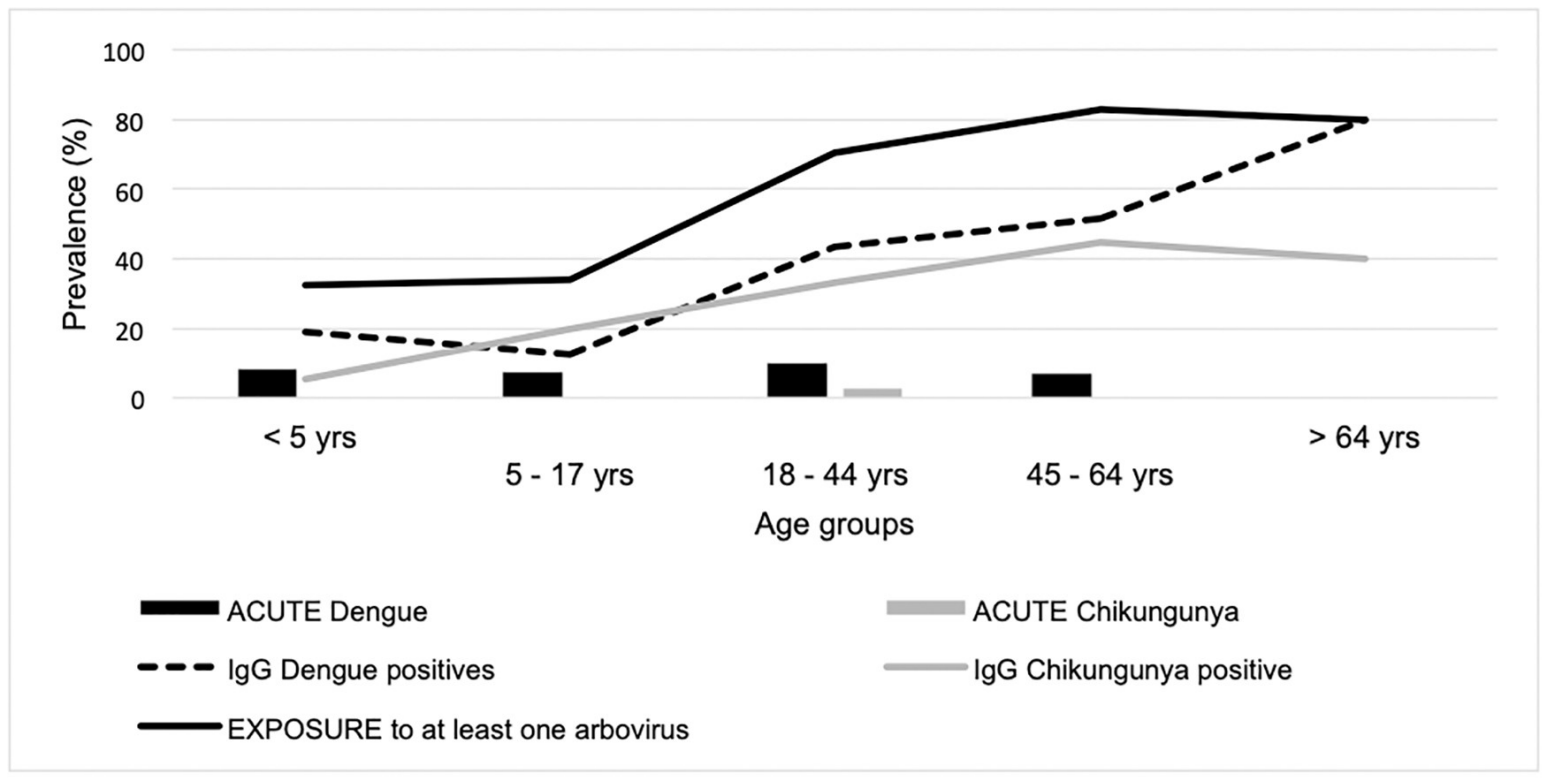
Prevalence of flavi- and alphaviruses infections and their related seroprevalence in patients with acute undifferentiated fever stratified by age at Lisungi health center, DRCongo, 11/2015-06/2016 (n = 235).

We found 71 (30.2%) patients with anti-DENV IgG antibodies, of which 60 (75.0%) contained relatively high levels of anti-DENV antibody (O.D. ≥ 1.9), the latter not depending on age (S1 Fig). The PRNT on the subsample was only positive in 5.6% and 66.7% for DENV, and 25% and 20% for YFV, on samples with IgG ratio below 1.9 and above 1.9,respectively (Table 3). For these past infections, all 4 serotypes were detected: DENV1, DENV2, DENV3 and DENV4 in 4, 2, 1 and 1 patient, respectively (of the 6 positively tested DENV PRNT)– 2 patients were reactive to all 4 serotypes, the other 4 only to 1 serotype. Past exposure to CHIKV was suspected with IFA IgG in 26.4% of the study participants. When taking the DENV and CHIKV positive IgG samples together, 56.6% of the study participants were suspected having been exposed to at least one arbovirus. The prevalence of past DENV and CHIKV infections increased with age, raising from 18.9% and 5.4% under 5 years of age, to 80% and 40% over 65 years of age, respectively (Fig 2). The association of age is statistically significant for the past infections with CHIKV (p = 0.01) and DENV (p<0.01) (S2 Table). Having been exposed to DENV was also statistically significant associated with recent travel (p = 0.01). The congruence between the RDT and PCR/ELISA results for DENV was variable: in comparison to PCR the sensitivity of NS1 was 90%, in comparison to IgM ELISA the IgM RDT had a sensitivity of 30% and in comparison to IgG ELISA the IgG RDT had a sensitivity of 7.6%. The specificities were all above 99.3%.

## Discussion

Although no large epidemics were reported recently, our study showed ongoing transmission of arboviruses in Kinshasa, DRC. Acute DENV, caused by DENV1 and DENV2, and CHIKV infection was demonstrated in 8.1% and 0.9% of the patients attending a first line health center with acute undifferentiated fever, respectively. Importantly, neither DENV nor CHIKV was clinically suspected, nor considered in the clinical differential diagnosis and 64.3% of patients were treated with at least one antibacterial drug of whom almost one in eight (11.7%) received dual or triple antimicrobial therapy. A possible explanation of the apparent absence of clinical and/or severe acute DENV cases in our study, and in other African settings too, is that African heritage is described to genetically protect against severe DENV. More specifically, the lower OSBPL10 expression profile in Africans is protective against viral hemorrhagic fever and dengue shock syndrome [36,37].

However, diagnostic testing for arboviruses has several shortcomings. Hereafter, we will highlight the limitations of the tests used for acute and past infections. Five out of 19 acute DENV and 2 out of 2 acute CHIKV infections were diagnosed based on the presence of IgM antibodies only and were therefore only presumptive infections. This could have led to an overestimation of the number of acute infections. In addition, IgM antibodies can be present for several months and it is therefore possible that DENV/CHIKV was not the cause of the fever at the moment of presentation. Nonetheless, the presence of IgM antibodies suggests that DENV/CHIKV was recently circulating in the area. On the other hand, as we did not have repeated measurements or convalescent samples to demonstrate seroconversion to IgG or a four-fold increase in IgG titer, we may have missed some acute secondary dengue cases, which may have undetectable IgM antibody levels [38].

The number of positive CHIKV IgM ELISA test results was unexpectedly high. As the results could not be confirmed with IFA, a technique which is considered to be more specific, interference with malaria was suspected based on our experience. Indeed, false-positive reactions as a result of polyclonal B-cell activation is a phenomenon that we experienced before with ZIKV ELISA [39]. The detection of *Plasmodium* by PCR in the CHIKV ELISA positive/IFA negative samples strongly supported this hypothesis. The use of IFA for IgM detection may result in false-positive reactions although the risk is limited in case of experienced readers [40]. Cross-reaction of anti-CHIKV antibodies with antibodies against other members of the Semliki-forest serogroup, notably O’nyong nyongvirus, could not be excluded as no neutralization assays were performed. ZIKV was not suspected to be circulating in the area and indeed, no RT-PCR positive cases were found. YFV was actively circulating in the region at the time of study, transgressing the border with Angola [22], but we did not detect any RT-PCR positive case. Detecting YFV and ZIKV only based on RT-PCR diagnostics could have resulted in an underestimation of acute ZIKV and YFV infections. However, ZIKV and YFV IgM testing was not done, because almost 80% of the patients included in this study presented in the first four days after the onset of fever, which is the period with the highest probability to detect the virus with molecular methods and there are several shortcomings with the IgM testing for these viruses [41,42]. Nowadays, there is evidence that in urine samples ZIKV is longer detectable by ZIKV PCR, but at the time of study this was not known. Thus, no such samples were collected [43].

We demonstrated past exposure to arboviruses too, with 30.2% of the participants having detectable IgG against DENV, which is on the higher end of the spectrum compared to other studies in Africa reporting an overall flavivirus seroprevalence ranging between 0 and 35% with a mean of 18.1% [9,25]. The IgG-seroprevalence increased with age, thus suggesting a continuous exposure to flaviviruses over time. The 26.4% past CHIKV infection rate was in line with the estimated seroprevalence of 34.4% in Congo Brazzaville before the outbreak of 2011 [20] and was on par with other African sites reporting an overall alphavirus seroprevalence oscillating between 0 and 72% [25]. Remarkably, CHIKV IgG was also detected in small children, born after the 2011 epidemic, pointing towards an endemic circulation of the virus. DENV IgG testing was done with an ELISA test. Although it is widely known that there is cross-reactivity among flaviviruses, ELISA is still the most affordable–hence most commonly used–test [25]. Since YFV vaccination is an expected cause of cross-reaction with DENV IgG, we performed PRNT for DENV and YFV in a subset of samples, and found that the majority of samples negative with PRNT for DENV, were also negative with PRNT for YFV, suggesting that the high flavivirus IgG positivity is not likely to be the result of YFV vaccination. Due to operational reasons and the scope of the study, we did not further test for other flaviviruses and hence, we cannot rule out that the DENV IgG positivity in our study is due to other flaviviruses exposure [44]. We noted that the congruence between DENV ELISA and PRNT was lower than in American and Asian settings [45–49], but similar to the observation in other studies in DRC [50] and in Ethiopia [51]. The report of YFV vaccination was very low (below 1%), but as YFV vaccination is included in the childhood vaccination program over the last decade, this may indicate that the population is not aware of which vaccines their children get. However, the increasing prevalence of flavivirus IgG antibodies with age is congruent with a history of increasing exposure to the pathogens over lifetime and thus could not be explained by YFV vaccination. The CHIKV IgG testing was done with two tests: screening with ELISA and confirmation with IFA. We cannot exclude that positive results are due to cross-reactivity with other known (e.g. O’nyong nyong virus, Semliki Forest virus or Sindbis virus) or unknown togaviruses.

In a recent study conducted at the Lisungi health center, it was reported that 62% of patients–both children and adults–with acute fever had neither malaria nor bacteremia [52]. For the first time we were able to demonstrate the fact that arboviruses, more specifically DENV and CHIKV, circulate in the capital of DRC. The highest number of acute cases was reported in June (a dry season month), but cases were also confirmed in the other months, indicating that despite an epidemic profile, transmission persists over the rainy season in Kinshasa. This finding is consistent with observations over the past decades in Asia [53] and Latin-America [54] and adds to mounting–although still scarce–evidence that arboviruses are endemic in large parts of sub-Saharan Africa [9,17].

Furthermore, we were able to document the common practice of over-prescription of antimicrobials, including antimalarial drugs, in malaria RDT-negative patients, as is apparently the case nationwide in DRC as recently shown by Ntamabyaliro et al [55]. Indeed, while not even half of the patients (45.3%) tested positive for malaria–a figure just below average national RDT positivity rates [30], close to 90% received antimalarial treatment, in addition to 15.5% of patients treated with over-the-counter antimalarials prior to presentation at the clinic. It could be questioned whether the rigorous implementation and usage of RDTs has any added benefit. A recent meta-analysis, including studies from Afghanistan, Cameroon, Ghana, Nigeria, Tanzania, and Uganda, evaluated data from over half a million children and adults and showed that the introduction of a malaria RDT simply shifted the antimicrobial overuse from one antimicrobial class to the other, mainly from antimalarial to antibacterial and anthelmintic drugs [56]. Consequently, the increasing prescription rate of antimicrobials–including antibacterial, anthelmintic and antimalarial drugs, is extremely worrisome in terms of the growing global problem of antimicrobial resistance, including against *Plasmodium falciparum* [57].

Although the sampling design of this study was adequate to evaluate the proportion of arboviruses causing acute undifferentiated fever, sample size was small and patients were only recruited from a single health center. However, the Lisungi health center is well visited by the surrounding population and all ages were represented in our study population. Moreover, the median age of our study population is 17 years, which approximates the median age of 18.6 years in the DRC (UNDESA 2017 and CIA World Factbook 2017). Another limitation in our study was the impossibility due to operational reasons to include participants over an entire year. The study was halted at the end of June, which was apparently the month with the highest number of infections. We did not investigate bacterial causes of fever through culture of blood or other bodily fluids, tests typically done in hospital settings, hereby possibly underestimating the burden of concomitant bacterial (super)infection. We therefore encourage further research elucidating the broad range of pathogens causing acute undifferentiated fever and the distribution of the insect vectors involved in arboviral transmission in urban and rural sub-Saharan African settings. Based on our findings, we recommend to include arboviral infections, namely DENV and CHIKV, in the differential diagnoses of acute fever presentation in Kinshasa.

In conclusion, we state that among undifferentiated acute fever cases in a peri-urban health center of Kinshasa, dengue–both DENV-1 and DENV-2 –and chikungunya infections were demonstrated, but no acute cases of Zika or yellow fever were detected. Apart from these acute infections, we showed that about one third of participants showed evidence of past arboviral exposure, as evidenced by positive IgG antibodies titers.

## Supporting information

S1 Text References for PCR-tests performed. (DOCX)

S1 Table Factors associated with acute dengue infection. (DOCX)

S2 Table Factors associated with past alpha- and flavivirus infection. (DOCX)

S1 Fig IgG titers of ELISA test for dengue according age. (TIF)

S1 Checklist STROBE Checklist. (DOCX)
