# Supplementary material for: Dengue and chikungunya among outpatients with acute undifferentiated fever in Kinshasa, Democratic Republic of Congo: A cross-sectional study
Source: PLoS Negl Trop Dis. 2019 Sep 5;13(9):e0007047. doi: 10.1371/journal.pntd.0007047 (PMC6748445; doi:10.1371/journal.pntd.0007047)
Supplement: S1 Text — (DOCX) [file pntd.0007047.s001.docx]

**Plasmodium PCR:**

Target: 18S rRNA

Amplicon: amplicon: 100-110 bp (species-dep)

ITM Antwerp protocol adapted from Rougemont et al. 2004, reference:

- Cnops L, Jacobs J, Esbroeck MV. Validation of a four-primer real-time PCR as a diagnostic tool for single and mixed Plasmodium infections. Clinical Microbiology and Infection. 2011;17: 1101–1107. doi:10.1111/j.1469-0691.2010.03344.x

**CHIKV PCR:**

Target: nSP1

Amplicon: 77bp

ITM Antwerp protocol adapted from Panning et al. 2008, references:

- Huits R, De Kort J, Van Den Berg R, Chong L, Tsoumanis A, Eggermont K, et al. Chikungunya virus infection in Aruba: Diagnosis, clinical features and predictors of post-chikungunya chronic polyarthralgia. Ansari AA, editor. PLOS ONE. 2018;13: e0196630. doi:10.1371/journal.pone.0196630
- Van Den Bossche D, Cnops L, Meersman K, Domingo C, Van Gompel A, Van Esbroeck M. Chikungunya virus and West Nile virus infections imported into Belgium, 2007–2012. Epidemiology and Infection. 2015;143: 2227–2236. doi:10.1017/S0950268814000685
- Panning M, Grywna K, van Esbroeck M, Emmerich P, Drosten C. Chikungunya fever in travelers returning to Europe from the Indian Ocean region, 2006. Emerging Infect Dis. 2008;14: 416–422. doi:10.3201/eid1403.070906

**DENV PCR:**

ITM Antwerp protocol adapted from Johnson et al. 2005 and Santiago et al. 2013, reference:

- Van den Bossche D, Cnops L, Van Esbroeck M. Recovery of dengue virus from urine samples by real-time RT-PCR. Eur J Clin Microbiol Infect Dis 2015; 34:1361–7.

**PDV PCR (for internal control extraction and PCR)** :

Target: H-protein (hemagglutinin) gene

Amplicon: 83bp

ITM Antwerp protocol adapted from Clancy et al. 2008, reference:

- Clancy A, Crowley B, Niesters H, Herra C. The development of a qualitative real-time RT-PCR assay for the detection of hepatitis C virus. Eur J Clin Microbiol Infect Dis. 2008;27: 1177–1182. doi:10.1007/s10096-008-0556-9

**ZIKV PCR**

Commercial Realstar Altona kit (Altona Diagnostics)
