# Supplementary material for: Dengue and chikungunya among outpatients with acute undifferentiated fever in Kinshasa, Democratic Republic of Congo: A cross-sectional study
Source: PLoS Negl Trop Dis. 2019 Sep 5;13(9):e0007047. doi: 10.1371/journal.pntd.0007047 (PMC6748445; doi:10.1371/journal.pntd.0007047)
Supplement: S1 Table — (DOCX) [file pntd.0007047.s002.docx]

**S1 Table: Factors associated with acute dengue infection**

|  |  |  | Univariate analysis | Multivariable analysis | |
| --- | --- | --- | --- | --- | --- |
|  | Total No tested (n=235) | Acute dengue N (%)  (n=19) | Crude OR (95% CI) | Adjusted OR (95% CI) ^$$^ | P value |
| Age groups:   - <5 year - 5-17 year - 18-44 year - ≥45 year | 37  80  81  34 | 3 (8.1)  6 (7.5)  8 (9.9)  2 (6.9) | REF  0.92 (0.22-3.89)  1.24 (0.31-4.97)  0.71 (0.11-4.52) | ^$^ |  |
| Gender:  - Female  - Male | 125  110 | 11 (8.8)  8 (7.3) | 1.23 (0.48-3.18)  REF | ^$^ |  |
| **Consulting during :**   - **June (dry season)** - **rainy season** | **41**  **194** | **10 (24.4 )**  **9 (4.6)** | **6.63 (2.49-17.62)**  **REF** | ^$^**6.13 (2.24-17.81)** | **<0.001** |
| Commune of residence:   - Lemba - Makala - Matete - Montngafula - Ngaliema - Selemboa | 1  1  1  175  23  34 | 0 (0)  0 (0)  0 (0)  16 (9.1)  2 (8.7)  1 (2.9) | -  -  -  3.32 (0.42-25.92)  3.14 (0.27-36.86)  REF |  |  |
| Recent travel  No travel | 5  230 | 1 (20.0)  18 (7.8) | REF  0.34 (0.04-3.20) |  |  |
| Yellow fever vaccination :   - Yes - no | 2  233 | 0 (0)  19 (8.2) | -  REF |  |  |
| Initial categorization at start of consultation:   - undifferentiated fever - gastroenteritis - urinary tract infection - acute respiratory infection | 191  7  12  25 | 13 (6.8)  0 (0)  1 (8.3)  5 (20.0) | 0.29 (0.09-0.90)  0  0.36 (0.04-3.52)  REF |  |  |
| Presence of chronic disease | 10 | 0 (0) | - |  |  |
| **Consultation within 4 days after onset of fever** | **183** | 15 (8.2) | 1.21 (0.38-3.86) |  |  |
| **Rash*** | **16** | 1 (5.9) | 0.69 (0.09-5.52) |  |  |
| **Muscular pain**** | **90** | **3 (3.3)** | **0.27 (0.08-0.96)** | ^$^**0.30 (0.08-1.15)** | **0.078** |
| **Painful or inflamed joints *** | **111** | 6 (5.3) | 0.44 (0.16-1.19) |  |  |
| Headache** | **163** | 12 (7.4) | 0.71 (0.27-1.90) |  |  |
| Tiredness** | 122 | 11 (9.0) | 1.30 (0.50-3.36) |  |  |
| Respiratory symptoms (upper and lower tract)* | 75 | 5 ( 6.3) | 0.67 (0.23-1.94) |  |  |
| Bleeding** | **1** | 0 (0) | - |  |  |
| Nausea** | **50** | 3 (6.0) | 0.67 (0.19-2.41) |  |  |
| Gastroenteral symptoms (vomiting, diarrhea, jaundice)* | **48** | 0 (0) | - |  |  |
| Abdominal pain** | **42** | 2 (4.8) | 0.52 (0.11-2.33) |  |  |
| Lymphadenopathy | **65** | **1 (1.5)** | **0.13 (0.02-1.01)** | ^$^**0.17 (0.02-1.35)** | **0.166** |
| Hepatomegaly and/or splenomegaly and/or abnormal abdominal palpation | **76** | **1 (1.3)** | **0.10 (0.01-0.78)** | ^$^0.17 (0.02-1.35) | 0.094 |
| Final diagnosis at consultation (> 1%):   - tonsillitis - unknown fever - malaria - other | 9  126  86  14 | 1 (11.1)  12 (9.5)  6 (7.0)  0 (0) | 1.67 (0.18-15.63)  1.4 (0.51-3.89)  REF  - |  |  |
| Requiring hospitalization | 32 | 0 (0) | - |  |  |
| Received treatment of at least one antibacterial drug | 157 | 11 (7.0) | 0.66 (0.25-1.71) |  |  |
| **Received treatment of more than one antibacterial drugs** | **26** | 2 (7.7) | 0.94 (0.20-4.33) |  |  |
| **Positive malaria Rapid Diagnostic Test** | **86** | 6 (7.0) | 0.94 (0.20-4.33) |  |  |
| Received an antimalarial treatment | 194 | 16 (8.2) | 0.73 (0.20-2.68) |  |  |
| Median Hematocrit (min-max) | 40 (14-55) | 40.5 (30-49) | 1.02 (0.94-1.10) |  |  |
| Median WBC count (min-max) | 4200 (2900-16300) | 4250 (3100-6600) | 1.00 (0.99-1.00) |  |  |
| **Median WBC formula (min-max)**   - neutrophils - lymphocytes - monocytes - eosinophils - basophils | 65 (28-94)  34 (6-72)  0 (0-4)  0 (0-3)  0 (0-1) | 67 (53-84)  31 (16-47)  0 (0-3)  0.5 (0-3)  0 (0-0) | 1.03 (0.96-1.10)  0.97 (0.91-1.04)  0.93 (0.52-1.65)  **2.39 (1.27-4.49)**  - |  |  |

*^$^entered in the backward (conditional) logistical regression model*

*^$$^only final model variables included*

**complaint by patient and/or observed during consultation*

***reported by patient*
