# Supplementary material for: Dengue and chikungunya among outpatients with acute undifferentiated fever in Kinshasa, Democratic Republic of Congo: A cross-sectional study
Source: PLoS Negl Trop Dis. 2019 Sep 5;13(9):e0007047. doi: 10.1371/journal.pntd.0007047 (PMC6748445; doi:10.1371/journal.pntd.0007047)
Supplement: S2 Table — (DOCX) [file pntd.0007047.s003.docx]

**S2 Table: Factors associated with past alpha- and flavivirus infection**

|  |  | **Past arbovirus** | | | | **Past alphavirus** | | | |
| --- | --- | --- | --- | --- | --- | --- | --- | --- | --- |
|  |  |  |  |  | |  |  |  | |
|  |  |  | Univariate analysis | Multivariable analysis | |  | Univariate analysis | Multivariable analysis | |
|  | Total No tested (n=235) | No positive  N (%)  N=71 | Crude OR (95% CI) | Adjusted OR (95% CI) | P value (multivariable analysis) | No positive  N (%)  N=62 | Crude OR (95% CI) | Adjusted OR (95% CI) | P value (multivariable analysis) |
| Age groups:   - <5 year - 5-17 year - 18-44 year - 45-64 year - ≥65 year | 37  80  81  29  5 | 7  10  35  15  4 | REF  0.61 (0.21-1.76)  3.26 (1.28-8.29)  4.59 (1.53-13.78)  17.14 (1.65-178.08) | REF  0.61 (0.20-1.89)  3.38 (1.23-9.27)  4.66 (1.43-15.11)  16.23 (1.48-177.58) | <0.01 | 2  16  27  13  2 | REF  4.37 (0.95-20.14)  8.75 (1.96-39.13)  14.22 (2.86-70.56)  11.67 (1.18-114.90) | REF  4.00 (0.86-18.63)  7.27 (1.61-32.88)  12.61 (2.51-63.49)  11.69 (1.12-121.56) | 0.01 |
| Gender:  - Female  - Male | 125  110 | 36  35 | 0.87 (0.50-1.51) |  |  | 31  31 | 0.84 (0.47-1.50) |  |  |
| **Consulting during:**   - **Dry season (June)** - **Rainy season** | **41**  **194** | 10  61 | 0.70 (0.32-1.53) |  |  | 12  50 | 1.19 (0.56-2.51) |  |  |
| Commune of residence:   - Lemba - Makala - Matete - Montngafula - Ngaliema - Selemboa | 1  1  1  175  23  34 | 0  0  0  49  5  17 | -  -  -  0.39 (0.18-0.82)  0.28 (0.08-0.92)  REF |  |  | 0  0  0  50  6  6 | -  -  -  1.87 (0.73-4.78)  1.65 (0.46-5.94)  REF |  |  |
| Recent travel  No travel | 5  230 | 4  67 | 9.73 (1.07-88.68) | 18.99 (1.80-199.88) | 0.01 | 1  61 | 0.69 (0.08-6.32) |  |  |
| Yellow fever vaccination :   - yes - no | 2  233 | 1  70 | 0.48 (0.03-7.77) |  |  | 0  62 | - |  |  |
| Requiring hospitalization | 32 | 9 | 0.89 (0.39-2.03) |  |  | 2 | 0.16 (0.04-0.69) | 0.20 (0.04-0.91) | 0.04 |
| Past arbovirus infection |  | _ | - |  |  | 19 | 1.03 (0.55-1.93) |  |  |
| Past chikungunya infection |  | 19 | 1.03 (0.55-1.93) |  |  | _ | - |  |  |
| Acute arbovirus infection |  | 0 | 0 |  |  | 8 | 2.18 (0.83-5.71) |  |  |
| Acute chikungunya infection |  | 1 | 2.33 (0.14-37.76) |  |  | 0 | - |  |  |
| Bleeding in current illness | **1** | 1 | - |  |  | 0 | - |  |  |
| Rash in current illness | **17** | 7 | 1.67 (0.61-4.59) |  |  | 4 | 0.84 (0.26-2.69) |  |  |
| Painful or inflamed joints | 117 | 43 | 1.87 (1.06-3.29) | 1.79 (0.95-3.40) | 0.072 | 31 | 1.01 (0.57-1.81) |  |  |
