# Supplementary figures and images for: Dengue and chikungunya among outpatients with acute undifferentiated fever in Kinshasa, Democratic Republic of Congo: A cross-sectional study
Source: PLoS Negl Trop Dis. 2019 Sep 5;13(9):e0007047. doi: 10.1371/journal.pntd.0007047 (PMC6748445; doi:10.1371/journal.pntd.0007047)

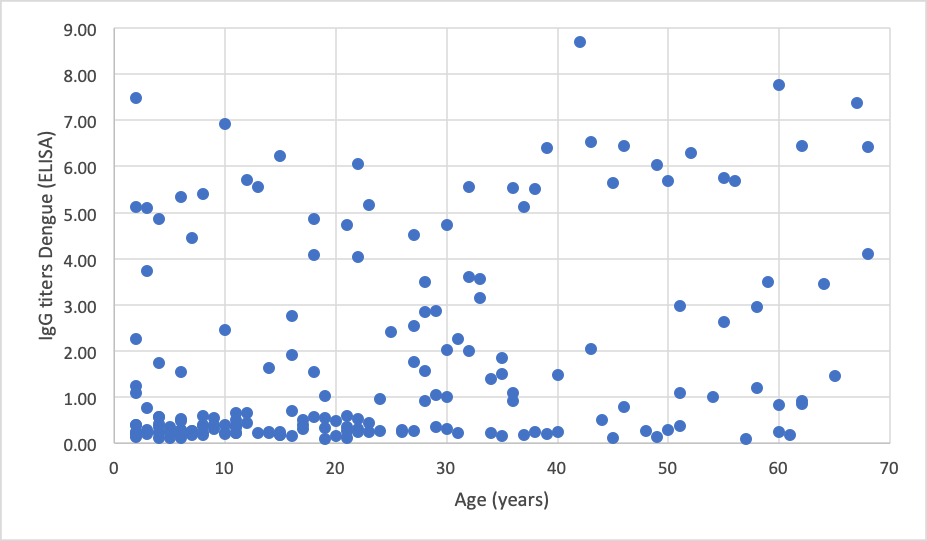

Supplement: S1 Fig — (TIF) [file pntd.0007047.s004.tif]
